# Future-Ready Doctors: A Cross-Sectional Study of Undergraduate Knowledge of Artificial Intelligence in Clinical Biochemistry

**DOI:** 10.7759/cureus.109794

**Published:** 2026-05-28

**Authors:** Susanna Theophilus Yesupatham, Ankita Kumari, Ravishankar Suryanarayana

**Affiliations:** 1 Biochemistry, Sri Devaraj Urs Medical College, Sri Devaraj Urs Academy of Higher Education and Research, Kolar, IND; 2 Biostatistics, Sri Devaraj Urs Medical College, Sri Devaraj Urs Academy of Higher Education and Research, Kolar, IND

**Keywords:** artificial intelligence, clinical biochemistry, healthcare technology, medical education, medical students

## Abstract

Background: Artificial intelligence (AI) is increasingly transforming healthcare delivery, particularly in laboratory medicine and clinical biochemistry. Despite its expanding applications, the preparedness of future medical professionals to engage with AI remains uncertain. Although several studies have evaluated awareness of AI among medical students globally, limited research has specifically focused on AI applications in clinical biochemistry and laboratory medicine in the Indian context. Aim: This study aims to appraise the knowledge, perceptions, and readiness regarding AI in clinical biochemistry among undergraduate MBBS students. Material and methods: A cross-sectional, questionnaire-based study was conducted over three months (September-December 2025) at Sri Devaraj Urs Medical College, following approval from the institutional ethics committee. A total of 268 MBBS students from Phase I to Phase III participated. Data was collected using a structured, self-administered Google form questionnaire (Google, Mountain View, CA, USA) covering demographics, knowledge, attitudes, readiness, and perceived barriers related to AI in clinical biochemistry. Data were analyzed using SPSS Statistics version 16 (IBM Corp. Released 2007. IBM SPSS Statistics for Windows, Version 16.0. Armonk, NY: IBM Corp.). Descriptive statistics were expressed as frequencies and percentages. Association between academic phase and responses was analyzed using the chi-square test, and effect size was assessed using Cramer’s V. A p-value of <0.05 was considered statistically significant. Results: Awareness of AI in healthcare among students was high (88.4%, n = 237); however, only 19% (n = 51) had received prior formal training in the use of AI. Knowledge of AI in clinical biochemistry increased progressively across academic phases, yet understanding of advanced laboratory applications remained limited. Students largely perceived AI as a supportive tool that enhances report accuracy, reduces errors, and improves laboratory turnaround time. The highest agreement was observed for the importance of AI knowledge in future medical practice (mean Likert score 4.06), whereas the concept of AI replacing laboratory professionals showed the lowest agreement (2.45). Ethical concerns such as data privacy and governance were widely recognized. More than half of the respondents (52.6%, n = 141) expressed willingness to undergo formal AI training. Conclusions: The findings highlight a significant gap between awareness and structured competency regarding AI among medical undergraduates. The need for early, phase-appropriate, and ethically grounded AI integration within the undergraduate medical curriculum is essential to prepare future physicians for an AI-enabled healthcare system.

## Introduction

Artificial intelligence (AI) refers to computational systems capable of performing tasks that typically require human intelligence, such as learning, reasoning, and problem-solving [1]. In modern healthcare systems, AI is increasingly applied across multiple domains, including diagnostic imaging, electronic medical records management, and laboratory automation [2]. Machine learning and data-driven analytical approaches have demonstrated substantial potential to improve diagnostic accuracy and reduce clinical errors.

Clinical biochemistry laboratories represent a major area where AI can be integrated effectively. AI-based algorithms can assist in interpreting laboratory results, identifying analytical errors, improving quality control systems, and also recognizing complex metabolic patterns associated with disease states [3]. During the pre-analytical phase, AI-assisted tools can identify inappropriate test requests, detect sample-labeling errors, and assess sample suitability for analysis [4]. In the analytical and post-analytical phases, AI supports large-scale data analysis, automated reporting, and risk stratification, which can ultimately enhance clinical decision-making and patient outcomes [5].

Limited formal education on AI concepts, inadequate technical exposure, and ethical concerns regarding data privacy and governance continue to pose barriers to adoption [6,7].

Undergraduate medical students represent the future healthcare workforce that will increasingly interact with AI-based technologies. Evaluating their knowledge, perceptions, and readiness is necessary for designing appropriate educational strategies. Previous studies suggest that although medical students generally express positive willingness toward AI, their foundational knowledge and technical competency remain limited [7,8].

In India, structured and formal AI training is not yet widely incorporated in medical curricula. Given the growing role of AI in laboratory medicine, it is important to assess medical students' awareness and readiness. Therefore, the present study aimed to evaluate MBBS students' knowledge, attitudes, perceptions, and preparedness regarding AI in clinical biochemistry and to identify educational gaps requiring targeted interventions [9].

The research abstract was presented orally at the Global Conference on Sustainable Materials and Smart Manufacturing, held from 5th to 6th January 2026, organized by Green Brigade Private Limited in Goa, India.

## Materials and methods

A cross-sectional questionnaire-based study was conducted over a period of three months (September-December 2025) at Sri Devaraj Urs Medical College, Kolar. Ethical approval was attained from the Central Ethics Committee of Sri Devaraj Urs Academy of Higher Education and Research (approval number: SDUAHER/R&D/CEC/SDUMC-PG/296/NF/2025-2026, approval date: 11/12/2025). Informed consent was collected digitally from all participants. The study included MBBS students from phases I, II, III part I, and III part II. A convenient sampling method was used.

For the inclusion and exclusion criteria, all students enrolled in phases I, II, and III who consented to participate in the study were included. At the same time, participants with incomplete questionnaire submissions were excluded.

Sample size was calculated based on the study by Preetha Jackson et al. using a 95% confidence level and 7% margin of error. The estimated minimum sample size obtained was 192. Considering feasibility and the need for adequate representation, the sample size was rounded to 200 participants [10].

Data were collected using a structured self-administered questionnaire distributed through Google Forms (Google, Mountain View, CA, USA). The questionnaire consisted of five sections: demographic details, which included age, gender, academic year/phase, and previous exposure to AI-related training; knowledge domain, which assessed students’ understanding of AI concepts, definitions, and applications in clinical biochemistry laboratories; attitude and perception, which evaluated perceived usefulness, reliability, and ethical considerations of AI using a 5-point Likert scale; future preparedness, which explored students’ willingness to learn AI, perceived opportunities, and potential challenges related to AI implementation in healthcare; and open-ended responses, which gathered students’ opinions regarding the usefulness and future implications of AI in medical practice.

The questionnaire was developed based on previously published studies assessing AI awareness among medical students and healthcare professionals [8,11,12]. The questionnaire was pilot tested among a small group of students to ensure clarity and relevance before final distribution. Content validity was assessed through expert review by faculty members in the Department of Biochemistry, and necessary modifications were incorporated prior to administration.

Data were entered into Microsoft Excel (Microsoft Corp., Redmond, WA, USA) and analyzed using SPSS Statistics version 16 (IBM Corp. Released 2007. IBM SPSS Statistics for Windows, Version 16.0. Armonk, NY: IBM Corp.). Descriptive statistics were expressed as frequencies and percentages. The association between the academic phase and responses was assessed using the chi-square test. Effect size was evaluated using Cramer’s V analysis. A p-value <0.05 was considered statistically significant.

## Results

A total of 268 students participated in the study. The demographic features of the participants are shown in Table 1.

**Table 1:** Demographic features of the participants (n = 268) AI: artificial intelligence, MBBS: Bachelor of Medicine and Bachelor of Surgery

Age in years	
17-20	175 (65.3%)
≥21	93 (34.7%)
Gender	
Male	82 (31%)
Female	186 (69%)
Phase of MBBS	
Phase I	118 (44%)
Phase II	53 (19.8%)
Phase III part I	36 (13.4%)
Phase III part II	61 (22.8%)
Have you previously attended any lecture, seminar, or workshop on AI?	
Yes	51 (19%)
No	217 (81%)

Demographic characteristics

Of the 268 participants, the majority of them (65.3%, n = 175) were 17 to 20 years of age. Female students constituted 69% (n = 186) of the study population, which reflects common gender distribution trends in medical education. Students were represented across all academic phases, allowing comparison between different stages of training. Notably, only 19% (n = 51) of participants reported prior exposure to formal AI training, while 81% (n = 217) had no formal educational experience related to AI.

Knowledge regarding AI and its applications in clinical biochemistry among undergraduate medical students across the phases of MBBS is shown in Table 2. Some values appeared similar across rows due to comparable response distributions among academic phases.

**Table 2:** Knowledge of AI and its applications in clinical biochemistry among undergraduate medical students across different phases of the MBBS program AI: artificial intelligence, MBBS: Bachelor of Medicine and Bachelor of Surgery

Variables student (n = 268)		Frequency n = 268	Phase I n = 118	Phase II n = 53	Phase III part I n = 36	Phase III part II n = 61	χ²	df	p-value	Cramer’s V
			%	%	%	%				
Have you heard about AI in healthcare?	Yes	237	40.3	21.6	14	24.23	9.84	3	0.020	0.19
	No	31	74.2	3.2	9.7	12.9				
Which of the following best defines AI?	A. Use of computers to mimic human intelligence and decision-making	214	42.7	20.7	13.1	23.5	18.67	9	0.028	0.15
	B. Basic computer programming for lab reports	26	50	23.1	3.8	23.1				
	C. Only robotics and automation	9	66.7	0	22.2	11.1				
	D. Not sure	19	42.1	10.5	26.3	21.1				
In which areas of clinical biochemistry laboratories can AI be applied?	A. Quality control and error detection	203	40.9	22.9	12.6	23.5	22.41	12	0.034	0.17
	B. Automated report generation	184	44.7	20.7	15.1	19.5				
	C. Pattern recognition in test results	170	42.7	22.2	15.2	19.9				
	D. Predictive diagnosis using biomarkers	148	44.4	18.9	14.4	19.8				
	E. I don’t know	17	35.3	23.5	17.6	23.5				
Which of the following AI-based tools do you think are already used in labs?	A. Automated analyzers	186	45.4	23.2	12.4	18.9	16.92	9	0.049	0.15
	B. Decision-support systems	52	45.3	24.5	3.8	26.4				
	C. Machine learning algorithms	237	46.2	18.3	21.2	32.7				
	D. Not sure	31	24	12	2	14				
AI can assist in	A. Reducing turnaround time	214	45.3	24.5	3.8	26.4	14.76	6	0.022	0.14
	B. Improving accuracy of lab results	26	53.7	12.2	24.4	12.2				
	C. Detecting abnormal values automatically	9	45.3	24.5	3.8	26.4				
	D. All of the above	19	45.4	23.2	12.4	18.9				

Participants were allowed to select multiple responses for applicable questionnaire items. Multiple responses were permitted for selected questionnaire items; therefore, cumulative percentages may exceed 100%.

Knowledge of AI

Overall awareness of AI in healthcare was high, with 88.4% (n = 237) of students reporting familiarity with the concept (p = 0.020). Approximately 74.2% (n = 199) correctly identified AI as algorithm-based systems capable of problem-solving and pattern recognition (p = 0.028).

Commonly recognized AI applications in clinical biochemistry included quality control and error detection (42.1%, n = 113), automated laboratory reporting (40.9%, n = 110), pattern recognition algorithms (46.2%, n = 124), and data analysis and interpretation (45.3%, n = 121). However, 15.3% (n = 41) of students reported no knowledge of AI applications in laboratory medicine (p = 0.049). Knowledge levels increased significantly across academic phases; however, the effect size was small, indicating only modest improvement in students' understanding of AI [13]. These findings suggest the need for structured AI education within the undergraduate medical curriculum to strengthen practical understanding of AI applications in clinical biochemistry.

Understanding of AI and its perceived use in clinical biochemistry using a 5-point Likert scale, as shown in Table 3.

**Table 3:** Understanding of AI and its perceived use in clinical biochemistry based on 5-point Likert scale scores AI: artificial intelligence

Variables	Response	Frequency	Percentage	Mean Likert score
AI can reduce human errors in biochemical reporting	Strongly agree	64	23.9	3.99
	Agree	142	53.0	
	Neutral	56	20.9	
	Disagree	6	2.2	
	Strongly disagree	0	0	
AI can completely replace medical laboratory professionals in the future	Strongly agree	18	6.7	2.45
	Agree	31	11.6	
	Neutral	64	23.9	
	Disagree	95	35.4	
	Strongly disagree	60	22.4	
AI can help in early disease detection by identifying hidden patterns in biochemical data	Strongly agree	59	22	3.95
	Agree	151	56.3	
	Neutral	46	17.2	
	Disagree	10	3.7	
	Strongly disagree	2	0.7	
AI can be more reliable than manual interpretation in detecting critical results	Strongly agree	39	14.6	3.42
	Agree	74	27.6	
	Neutral	118	44	
	Disagree	34	12.7	
	Strongly disagree	3	1.1	
Understanding AI is important for the future of clinical medicine	Strongly agree	79	29.5	4.06
	Agree	132	49.3	
	Neutral	51	19	
	Disagree	5	1.9	
	Strongly disagree	1	0.4	
AI use in labs raises concerns about ethical issues, data privacy, and patient confidentiality	Strongly agree	65	24.1	3.91
	Agree	126	47	
	Neutral	64	23.9	
	Disagree	11	4.1	
	Strongly disagree	2	0.7	

Attitudes and perceptions

Students generally demonstrated a positive attitude toward AI in healthcare. The mean Likert score for AI reducing human error in laboratory practice was 3.99; for AI assisting in early disease detection, 3.95; for AI improving the reliability of laboratory reports, 3.87; and for knowledge of AI being essential for future medical practice, 4.06. Ethical concerns regarding data privacy and governance were also acknowledged, with a mean score of 3.91. The lowest agreement was observed for the statement that AI could replace laboratory professionals, with a mean score of 2.45. These findings suggest that students view AI primarily as a supportive tool rather than a replacement for healthcare professionals, consistent with previous literature [6,12].

Confidence and readiness

**Figure 1:**
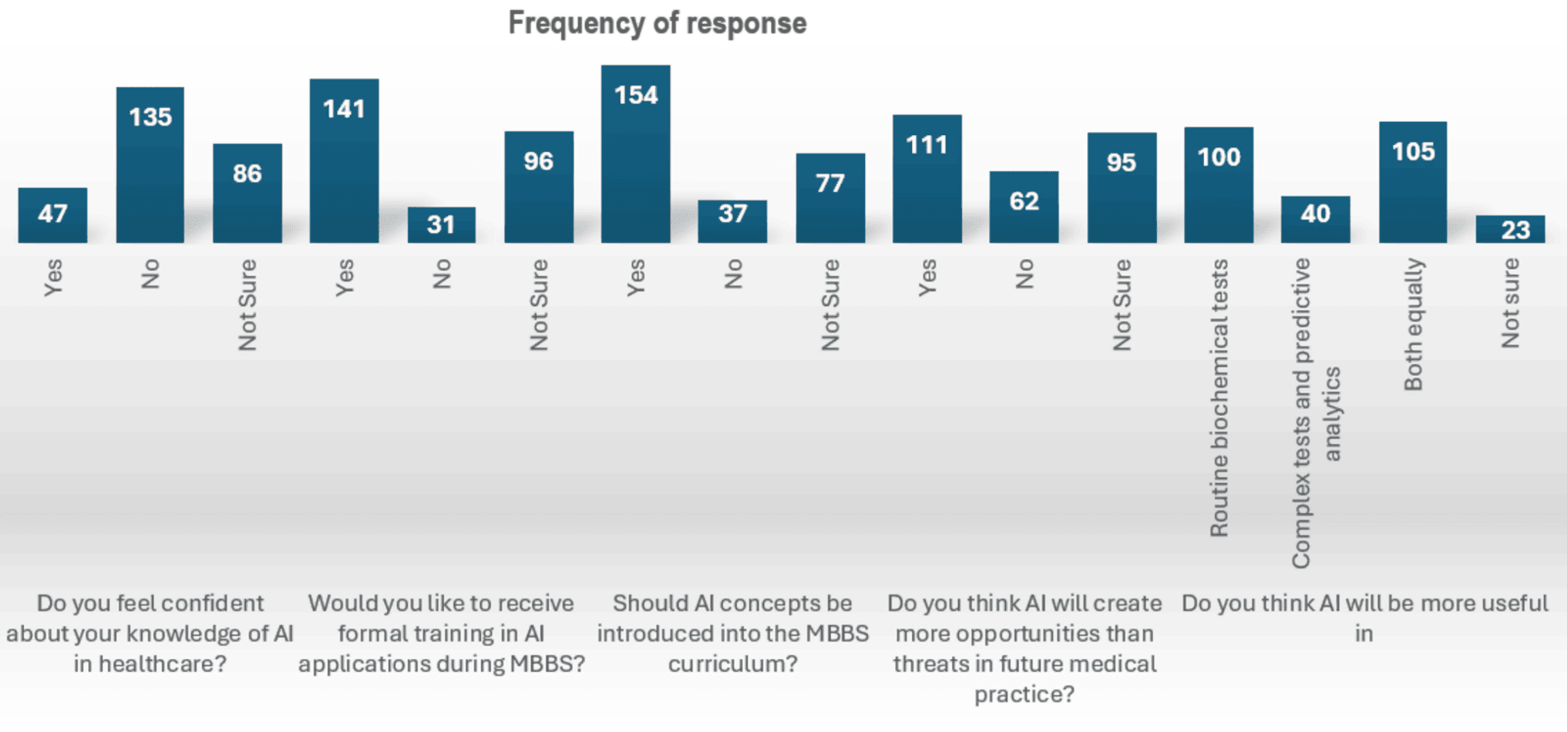
Attitudes and future prospects in adopting AI in healthcare AI: artificial intelligence, MBBS: Bachelor of Medicine and Bachelor of Surgery

Only 18% (n = 48) of students reported confidence in their knowledge of AI. Around 50.4% (n = 135) expressed uncertainty, and 32.1% (n = 86) remained neutral regarding their competency. More than half of the participants (52.6%, n = 141) indicated willingness to receive structured AI training. Preferred learning formats included workshops and training sessions preferred by 50% (n = 134) of students, integration into medical curriculum by 29.9% (n = 80), and online courses and digital platforms preferred by 20.2% (n = 54) of the students, as shown in Table 3 and Figure 1.

Opportunities and barriers in adopting AI

Around 41.4% (n = 111) of students believed AI would create more opportunities than risks in healthcare, while 35.4% (n = 95) remained uncertain. Perceived barriers included lack of technical expertise among 29.5% (n = 79) of students, high implementation costs among 29.1% (n = 78), data privacy concerns among 17.9% (n = 48), and ethical issues among 15.3% (n = 41) of students. Overall, students demonstrated a positive attitude toward AI adoption in healthcare but limited confidence in their knowledge, highlighting the need for formal educational interventions [14,15].

## Discussion

The present study demonstrated that although MBBS students possess general awareness of AI, their knowledge remains limited and lacks structured educational support. Similar findings have been reported, which indicate that medical students generally show a favorable attitude toward AI despite limited structured AI training [8,16,17].

Although knowledge scores showed a statistically significant increase across academic phases, the small effect size suggests that the improvement in AI understanding was modest. However, the absence of a formal AI curriculum limits the development of deeper competencies required for its effective integration into clinical practice.

Students primarily associated AI with automation and error reduction in laboratory processes, reflecting a practical but limited conceptual understanding. Ethical concerns, particularly regarding data privacy and governance, were widely recognized and aligned with global debates on responsible AI implementation in healthcare [18].

Interestingly, most students rejected the notion that AI would replace laboratory professionals. Instead, they viewed AI as a supportive technology that enhances human decision-making, a perspective supported by prior research [6,19].

Students expressed strong interest in structured AI education, underscoring the growing recognition of its relevance to future medical practice. Integrating AI-related modules, including practical exposure, interdisciplinary workshops, and ethical training, into the undergraduate medical curriculum will ensure that future physicians are adequately prepared for technology-driven healthcare systems [3,20,21].

In the open-ended responses, students commonly highlighted the need for structured AI training, practical workshops, and guidance regarding ethical and responsible AI use in healthcare. Several participants also expressed concerns about data privacy, the reliability of AI-generated results, and the potential overreliance on AI tools in clinical practice.

Limitations of the study

This was a single-institution study. A convenience sampling method based on voluntary participation was used, which may not be representative of the entire undergraduate medical student population. In addition, longitudinal follow-up was not included, which could have helped determine whether educational interventions and increased exposure to AI concepts improve medical students’ knowledge and readiness.

## Conclusions

Undergraduate MBBS students demonstrate substantial awareness of AI in healthcare; however, formal learning and confidence in applying AI concepts in clinical biochemistry remain limited. Students largely perceive AI as a supportive technology that can enhance diagnostic accuracy and laboratory efficiency while recognizing ethical considerations. These findings support the recommendation to incorporate structured, ethically guided AI education into undergraduate medical curricula to improve awareness and preparedness for AI applications in healthcare.
